# Could fertility-sparing surgery be considered for stage I ovarian sex cord-stromal tumors? A comparison of the Fine-Gray model with Cox model

**DOI:** 10.3389/fonc.2022.964181

**Published:** 2022-09-08

**Authors:** Dan Sun, Zhi F. Zhi, Jiang T. Fan

**Affiliations:** Department of Gynecology and Obstetrics, The First Affiliated Hospital of Guangxi Medical University, Nanning, China

**Keywords:** Fertility-sparing surgery, ovarian, sex cord-stromal tumors, Fine-Gray model, cancer-specific survival, Cox model, overall survival

## Abstract

**Objective:**

To evaluate the oncologic outcomes in patients with International Federation of Gynecology and Obstetrics (FIGO) stage I ovarian sex cord-stromal tumors (SCSTs) who underwent fertility-sparing surgery (FSS) and the independent risk factors affecting overall survival (OS) and cancer-specific survival (CSS).

**Methods:**

Data were acquired from the Surveillance, Epidemiology, and End Results (SEER) database between 1973 and 2018. A total of 240 patients diagnosed with stage I ovarian SCSTs were divided into the definitive surgery group (N=116) and FSS group (N=124). The Kaplan-Meier analysis and Cox model were used to evaluate the overall survival (OS) and cancer-specific survival (CSS) of the two groups and assess the independent risk factors respectively. The Fine-Gray model evaluated cancer-specific mortality (CSM) and the independent risk factors that affected CSM.

**Results:**

Kaplan-Meier survival analysis showed no statistically significant differences in OS and CSS between the two groups (P>0.05). Univariate analysis of the Fine-Gray model also showed that there was no difference in the CSS between the two groups (P>0.05). However, from the 15th year postoperatively, the CSS of the FSS group decreased by 13.21% compared with that of the control group and by 17.49% in the 20th and 25th years postoperatively. The Cox proportional hazards model found that surgical methods (“defined surgery” vs “FSS”; HR=0.03259, P=0.0196) and FIGO stage (“stage IA” vs “stage IC”; HR=0.03073, P=0.0300) were independent risk factors for OS. The multivariate analysis of Fine-Gray model showed that the cancer-specific mortality of patients receiving definitive surgery was 40.1% lower than that of patients receiving FSS (“definitive surgery” vs “FSS”; HR=0.599, P=0.005), indicating that FSS might lead to higher tumor-specific mortality and lower CSS. However, age, race, laterality, history, FIGO stage, and tumor size had no significant influence on the tumor-specific mortality (P>0.05).

**Conclusions:**

FSS is considered for patients with stage I SCSTs with reproductive needs, but the follow-up period should not be less than 15 years. For patients with stage IC disease, FSS should be selected carefully, and close follow-up is necessary. Perhaps, definitive surgery after birth is a means to improve long-term survival rates.

## Introduction

Women of childbearing age account for approximately 12% of patients with malignant ovarian tumors (1). In patients with malignant ovarian tumors who have reproductive needs, not only should we pay attention to the outcome of tumor treatment, but also the quality of life, and fertility retention should not be ignored. Fertility-sparing surgery (FSS) for malignant ovarian tumors aims to preserve the normal ovaries and/or uterus of patients (2). To ensure the therapeutic effect on tumors, the reproductive endocrine and reproductive functions of patients should be preserved (2).

Sex cord-stromal tumors (SCSTs) are rare ovarian non-epithelial tumors, accounting for approximately 2–5% of ovarian malignant tumors (3). Moreover, 60%–95% of patients are diagnosed as International Federation of Gynecology and Obstetrics (FIGO) stage I (4). Patients with stage 1 have a good prognosis, with a recurrence risk of ≤ 5% and a 5-years cancer-specific survival (CSS) of 98% (5, 6). Granulosa tumors and Sertoli-Leydig cell tumors are common pathological types of SCSTs (4). Among them, granulosa tumors account for approximately 70%, which has a tendency for late recurrence (4). Given that the majority of patients with SCSTs have an an indolent course, a long-term follow-up is needed (3). A research suggested close surveillance might be an alternative option to avoid chemotherapy for some stage I patients with malignant ovarian germ cell tumors (MOGCT) (7). For patients who have undergone FSS, physical exam, tumor marker detection and pelvic ultrasound must be carried out every 6 months beginning from the third year after surgery (4, 8). For patients with stage I SCSTs who have fertility needs, FSS is an acceptable approach (8). It is feasible to perform FSS for those who wish to preserve their reproductive function and whose tumors are confined to the ovaries, and all other patients are advised to undergo definitive surgery (8). Therefore, definitive surgery is the standard treatment for stage I SCSTs.

The prognosis of patients with SCST after FSS remains controversial. Presently, multicenter and large-sample prospective studies to confirm the safety of FSS are lacking, and most of these are retrospective studies. A retrospective study found that the 5-year overall survival (OS) of patients with stage I granulosa tumor who underwent FSS was lower than that of patients who underwent definitive surgery (87.7% vs 92.6%, P<0.001) (9). Some studies also reported that, compared with patients with stage I granulosa tumors who underwent definitive surgery, patients who received FSS had worse 10-year disease-free survival (50% vs 74%, P = 0.006) (10). A retrospective study based on the Surveillance, Epidemiology, and End Results (SEER) database in 2017 found that the 20-year CSS of patients with stage I premenopausal SCSTs who underwent FSS was lower than that of patients who underwent definitive surgery (94.2% vs 71.7%, P = 0.021) and pointed out that FSS was associated only with a worse long-term CSS compared with definitive surgery (11).

However, clinical survival data are often accompanied by multiple outcomes, in which there may be a competitive relationship. These studies did not consider the occurrence of competitive events. Ignoring the competitive risk, the traditional Kaplan-Meier analysis will overestimate the cumulative mortality rate, and the Cox model may misjudge the hazard ratio (12). Therefore, based on the SEER database, the Fine-Gray model was used to explore the influence of FSS and other clinical parameters on the prognosis of patients with stage I SCSTs. The results of the Kaplan-Meier analysis, Cox model, and Fine-Gray model were compared. This is also the first study to evaluate the influence of FSS on the prognosis of patients with stage I SCSTs by Fine-Gray model.

## Methods

### Data source and patient collection

In this retrospective study, we used SEER*Stat software version 8.3.9.2 to download 270 cases of ovarian SCSTs from the SEER database between 1973 and 2018. The database was jointly established by 18 registration authorities in various states and regions of the United States and is one of the most representative large-scale tumor registration databases in North America. A large amount of relevant evidence-based medicine data were collected. The data were downloaded with permission from the relevant institutions. The inclusion criteria were as follows: age ≤ 49 years; ovary as the tumor site (ICD-O-3/WHO2008, C56.9 ovary); SCSTs as the histological type confirmed by pathology; and FIGO stage I (tumor confined to the ovary). The exclusion criteria were as follows: follow-up time < 1 month, unknown pathology (codes 00, 10, 17), unknown surgical method (codes 35, 50, 55, 90); and death (missing/unknown).

**Figure 1:**
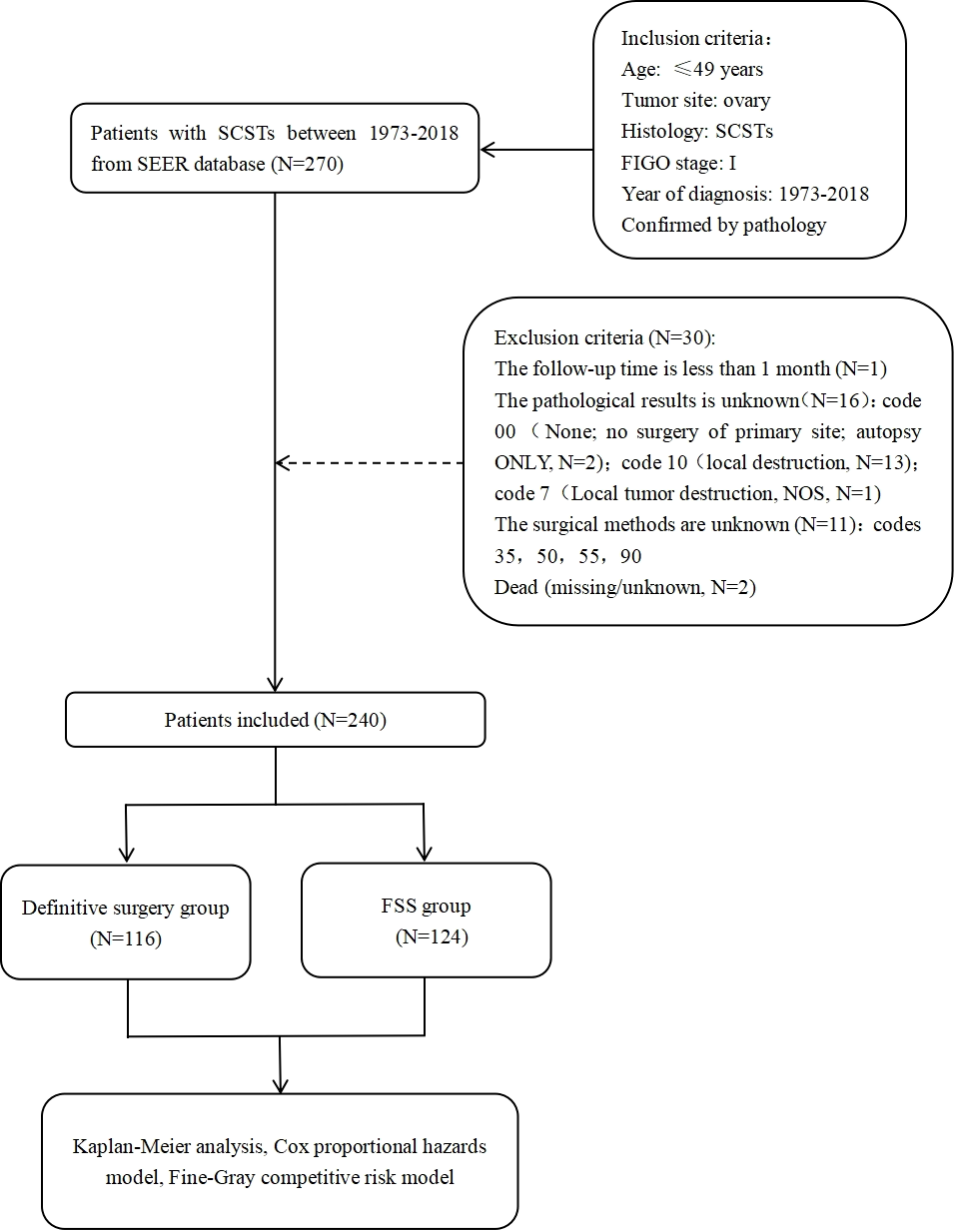
Flow chart of participants enrollment. SCSTs, sex cord-stromal tumors; FIGO, International Federation of Gynecology and Obstetrics; NOS, not otherwise specified; FSS, fertilitysparingsurgery.

According to these criteria, 240 patients with stage I ovarian SCSTs were included. According to the surgical method, the patients were divided into the definitive surgery group (control group, N=116) and FSS group (case group, N=124). The surgical methods of SCSTs mainly include definitive surgery/FSS and comprehensive staging operation (careful exploration, biopsies of all suspicious sites, local cytoreductive surgery) (4). There is no consensus of nodal debulking surgery considering the low risk of nodal spread of germ cell tumours (GCT) (4). In our research, definitive surgery included bilateral salpingo-oophorectomy and hysterectomy. FSS was defined as unilateral salpingo-oophorectomy, bilateral salpingo-oophorectomy, or tumor resection without hysterectomy. The screening process for the study population is presented in **Figure 1**.

### Study variables

The variables included in this study were as follows age, year of diagnosis, race, laterality, history, stage, tumor size (mm), follow-up time (months), and endpoint events. The exact definitions of these variables can be found on the SEER database website (https://seer.cancer.gov/). Overall survival (OS) and cancer-specific survival (CSS) are evaluation indexes of tumor survival, but CSS was the primary outcome of this study. When comparing the Fine-Gray model with Kaplan-Meier analysis and Cox model, CSS was the only evaluation index. OS was defined as the interval from the date of surgery to the date of death from any cause or the last follow-up. CSS was defined as the survival time from the patient’s diagnosis of SCSTs to death specifically attributable to SCSTs. In the univariate analysis of the Fine-Gray model, CSS was equal to 1 minus cancer-specific mortality (CSM). Death caused by SCSTs was considered an interesting event. Loss to follow-up and survival by the end of 2017 were regarded as censored events. The events leading to nonspecific death in stage I SCSTs were considered competitive events.

Based on age at diagnosis, there were two age groups: 5–34 years and 35–49 years.

Based on year of diagnosis, the patients were divided into the 1988–1997 group, 1998–2007 group, and 2008-2017 group.

Based on race, patients were divided into white, black, others, and unknown.

Based on laterality, patients were divided into left, right, and unknown.

The ICD-0-3 site/histology validation list from code 8590/3 to code 8670/3 was used to distinguish granulosa and nongranulosa. Nongranulosa mainly includes Sertoli-Leydig cell tumor, thecoma, granulosa cell-theca cell tumor, steroid cell tumor, and others.

All patients had FIGO stage I; that is, the tumor was confined to the ovary, including IA, IB, IC, I, or not otherwise specified (NOS).

According to the tumor diameter, we divided the tumor size into ≤ 100 mm, 101–200 mm, >200 mm, and unknown.

The follow-up time was divided into ≤ 60 months, 61–120 months, 121–240 months, and > 240 months.

The endpoint events include survival, death of ovarian cancer, and death of other causes.

### Statistical analysis

Comparisons of OS and CSS between the FSS and definite surgery groups were performed using Kaplan-Meier analysis, and the differences between the curves were analyzed using the log-rank test. Multivariate Cox regression models were used to estimate the hazard ratios to analyze the independent prognostic factors associated with OS and CSS in patients with stage I SCSTs. According to the causes of death recorded in the database, patients who died from non-tumor causes were classified into two categories: those who died of SCSTs and those who died of non-SCSTs. The Fine-Gray model was used to explore the factors influencing the prognosis of patients with stage I SCSTs, which showed an effect based on the subhazard ratio. Then, the cumulative incidence function (CIF) was used to calculate the trend of the cumulative incidence of death in each end event with time in the presence of competitive events. All statistical analyses and graph construction were performed using R software (version 4.1.3; www.r-project.org). The P-values were two-sided, and a P-value < 0.05 was considered statistically significant.

## Results

### Demographic and patient characteristics

Finally, we included 240 patients with stage I SCSTs, including 116 (48.33%) in the definitive surgery group and 124 (51.67%) in the FSS group, all of whom were distributed between 1988 and 2017. The demographic and clinical characteristics of the patients with stage I SCSTs who underwent different surgical methods are shown in **Table 1**. The patients in the FSS group were younger than those in the control group. Of the patients, 58.87% were aged 5-34 years, whereas 87.93% of the patients in the definitive surgery group were aged 35-49 years. Granulosa was the main pathological type in both groups, accounting for 77.08% of the total patients. The FIGO stage of patients was mainly concentrated in stages IA and IC, while stage IB only occurred in one case in the control group, so the statistical analysis only included patients with stages IA and IC. Patients in both groups were mainly white and distributed between 2008 and 2017 and had tumors ≤ 200 mm, accounting for the highest proportion. The average follow-up time of the definitive surgery group was 143.09 ± 8.42 months, while that of the FSS group was 100.85 ± 6.20 months. A total of 270 (87.50%) patients survived, and 18 patients (7.50%) eventually died of ovarian cancer, including eight patients in the control group (6.90%) and 10 patients in the case group (8.06%). There were significant statistical differences between the two groups in terms of age at diagnosis, year of diagnosis, race, history, follow-up time, and endpoint event (P < 0.05). The indexes with no statistical difference were laterality, FIGO stage, and tumor size (P > 0.05, **Table 1**).

**Table 1:** Demographic data and independent variable distribution of 240 patients with stage I SCSTs between the definitive surgery group and FSS group.

Variate	Category	Total (%, N = 240)	Definitive surgery (%, N = 116)	FSS (%, N = 124)	*P*-value
Diagnosis age (years)	5–34	87 (36.25)	14 (12.07)	73 (58.87)	0.000
	35–49	153 (63.75)	102 (87.93)	51 (41.13)	
Year of diagnosis	1988–1997	45 (18.75)	32 (27.59)	13 (10.48)	0.000
	1998–2007	66 (27.50)	40 (34.48)	26 (20.97)	
	2008–2017	129 (53.75)	44 (37.93)	85 (68.55)	
Race	White	150 (62.50)	76 (65.52)	74 (59.68)	0.045
	Black	66 (27.50)	26 (22.41)	40 (32.26)	
	Others	21 (8.75)	14 (12.07)	7 (5.65)	
	Unknown	3 (1.25)	0 (0.00)	3 (2.42)	
Laterality	Left	127 (52.92)	62 (53.45)	65 (52.42)	0.325
	Right	111 (46.25)	52 (44.83)	59 (47.58)	
	Unknown	2 (0.83)	2 (1.72)	0 (0.00)	
Histology	Granulosa	185 (77.08)	98 (84.48)	87 (70.16)	0.008
	Nongranulosa	55 (22.92)	18 (15.52)	37 (29.84)	
FIGO stage	IA	180 (75.00)	90 (77.59)	90 (72.58)	0.416
	IB	1 (0.42)	1 (0.86)	0 (0.00)	
	IC	50 (20.83)	20 (17.24)	30 (24.19)	
	I,NOS	9 (3.75)	5 (4.31)	4 (3.23)	
Tumor size (mm)	≤ 100	92 (38.33)	51 (43.97)	41 (33.06)	0.296
	101–200	74 (30.83)	30 (25.86)	44 (35.48)	
	> 200	27 (11.25)	13 (11.21)	14 (11.29)	
	Unknown	47 (19.58)	22 (18.97)	25 (20.16)	
Follow-up time (months)	≤ 60	58 (24.17)	21 (18.10)	37 (29.84)	0.000
	61–120	84 (35.00)	33 (28.45)	51 (41.13)	
	121–240	71 (29.58)	41 (35.34)	30 (24.19)	
	> 240	27 (11.25)	21 (18.10)	6 (4.84)	
	Mean ± SD	121.27 ± 5.34	143.09 ± 8.42	100.85 ± 6.20	
	Q1, Q3	62.25, 166.50	26.5, 204.25	51.25, 132.00	
Endpoint event	Survival	210 (87.50)	98 (84.48)	112 (90.32)	0.044
	Death of ovarian cancer	18 (7.50)	8 (6.90)	10 (8.06)	
	Death of other causes	12 (5.00)	10 (8.62)	2 (1.61)	

FSS, fertility-sparing surgery; FIGO, International Federation of Gynecology and Obstetrics; NOS, not otherwise specified

### Kaplan-Meier analyses of surgical methods for OS and CSS

**Figure 2:**
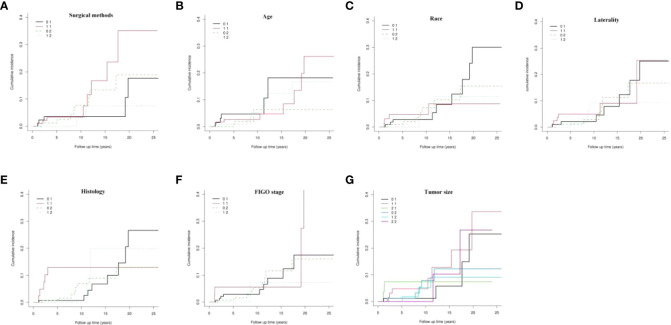
Kaplan-Meier survival analysis of definitive surgery group and FSS group. **(A)** overall survival (OS), **(B)** cancer-specific survival (CSS); FSS, fertility-sparing surgery.

Kaplan-Meier survival analysis showed that, generally, there was no statistical difference in postoperative OS (P = 0.786, **Figure 2A**) and CSS (P = 0.122, **Figure 2B**) between patients who underwent definitive surgery and those who underwent FSS.

### Cumulative incidence of death in two end events analyzed by Fine-Gray model

**Figure 3:**
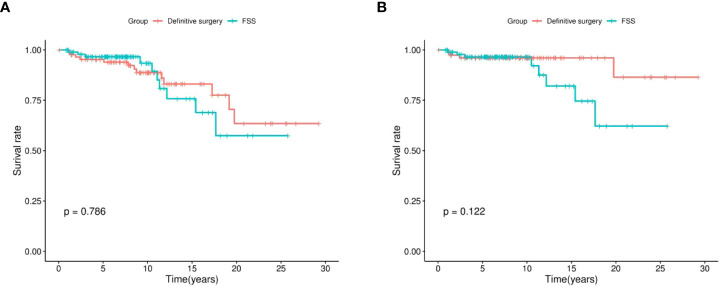
Cumulative incidence of death in two end events. FIGO, International Federation of Gynecology and Obstetrics.

To eliminate the influence of competitive events, we used the CIF to draw the curves of the dynamic change in the cumulative incidence of death in the two end events (**Figure 3**). The results showed that there was generally no significant difference in the cumulative incidence of death for each variable between the two end events (P > 0.05, **Figure 3A-G**).

However, through further analysis of the data of patients who underwent definitive surgery, we found that the CSM rates at the 5th, 10th, 15th, 20th, and 25th years postoperatively were 3.53%, 3.53%, 3.53%, 17.63%, and 17.63%, respectively. Moreover, the CSS in the 5th, 10th, 15th, 20th, and 25th years postoperatively were 96.47%, 96.47%, 82.37%, and 82.37%, respectively. For those who preferred FSS, the CSM rates at the 5th, 10th, 15th, 20th and 25th years postoperatively were 3.38%, 3.38%, 16.74%, 35.12%, and 35.12%, respectively. Furthermore, the CSS at the 5th, 10th, 15th, 20th, and 25th years postoperatively were 96.62%, 96.62%, 83.26%, 64.88%, and 64.88%, respectively. In the first 10 years after surgery, the CSS of the two groups was similar, but from the 15th year after surgery (83.26% vs 96.47%), the CSS of patients in the FSS group began to decrease by 13.21% compared with that of the control group. At the 20th year (64.88% vs 82.37%) and 25th year (64.88% vs 82.37%) after surgery, the CSS in the FSS group decreased by 17.49% compared with the definitive surgery group.

### Identification of prognostic factors of oncologic outcomes

To eliminate the influence of confounding factors and identify the independent risk factors that affect the prognosis of patients, multivariate Cox regression was used to analyze the factors associated with OS and CSS in patients with stage I SCSTs. As shown in **Table 2**, the surgical method was an independent risk factor of OS (“definitive surgery” vs “FSS”; HR = 0.03259, P = 0.0196), but not an independent risk factor of CSS (HR = 3.0196, P = 0.1220). The results showed that the OS of patients who underwent FSS was shorter than that of those who underwent definitive surgery. In terms of OS, Cox analysis also showed that FIGO stage was a prognostic factor (“stage IA” vs “stage IC”; HR = 0.03073, P = 0.0300). That is, patients with stage IC disease had a shorter OS. Moreover, histology is an independent risk factor of CSS (“granulosa” vs “nongranulosa”; HR = 4.7618, P = 0.0235). Granulosa was associated with worse CSS than nongranulosa. The results also indicated that age at diagnosis, race, laterality, and tumor size were not related to both OS and CSS in the cohort (**Table 2**).

**Table 2:** Independent risk factors affecting OS and CSS analyzed by Cox proportional hazards model.

Variables	OS				CSS			
	HR	HR. 95L	HR. 95H	*P*-value	HR	HR. 95L	HR. 95H	*P*-value
**Surgical methods**	0.0326	0.0018	0.5779	0.0196	3.0196	0.7442	12.2526	0.1220
**Age at diagnosis**	0.1624	0.0085	3.0976	0.2269	2.4895	0.5532	11.2035	0.2346
**Race**	29.0689	0.8421	1003.4201	0.0622	1.4535	0.7783	2.7144	0.2406
**Laterality**	2.5718	0.3058	21.6288	0.3846	1.4358	0.4428	4.6556	0.5468
**Histology**	0.5531	0.0719	4.2541	0.5694	4.7618	1.2339	18.3764	0.0235
**FIGO stage**	0.0307	0.0013	0.7133	0.0300	2.9263	0.8308	10.3063	0.0946
**Tumor size**	0.2089	0.0297	1.4669	0.1153	1.2942	0.5948	2.8158	0.5156

HR, hazard ratio; OS, overall survival; CSS, cancer-specific survival; FIGO, International Federation of Gynecology and Obstetrics.

Considering the influence of competitive events, we used multivariate analysis of the Fine-Gray model to explore the independent risk factors that affect prognosis. As shown in **Table 3**, except for surgical methods (P = 0.005), age at diagnosis, race, laterality, history, FIGO stage, and tumor size had no significant influence on CSM (P > 0.05). Among them, the CSM of patients who underwent definitive surgery decreased by 40.1% compared with those who underwent FSS (“definitive surgery” vs “FSS”; HR = 0.599, P = 0.005). Thus, FSS can increase the CSM and decrease the CSS rates in patients with stage I SCSTs. Unlike the Cox model (**Table 2**), FIGO stage is no longer an independent risk factor for prognosis after eliminating the interference of competitive events (“IA” vs “IC”; HR = 0.725, P = 0.134, **Table 3**).

**Table 3:** Multivariable analysis of prognostic factors in patients with stage I SCSTs using the Fine-Gray model.

Variables	HR	St.Err.	t value	HR. 95L	HR. 95H	*P*-value
**Surgical methods**	0.599	0.109	-2.81	0.419	0.856	0.005
**Age at diagnosis**	0.981	0.204	-0.09	0.653	1.474	0.926
**Race**	0.865	0.148	-0.85	0.619	1.210	0.397
**Laterality**	0.869	0.142	-0.86	0.631	1.196	0.388
**Histology**	0.807	0.192	-0.90	0.506	1.287	0.367
**FIGO stage**	0.725	0.156	-1.50	0.475	1.104	0.134
**Tumor size**	1.032	0.112	0.29	0.835	1.276	0.771

HR, hazard ratio; FIGO, International Federation of Gynecology and Obstetrics.

## Discussion

This is the first study to use the Fine-Gray competitive risk model in the evaluation of the influence of FSS and other parameters on the prognosis of patients with stage I SCSTs. FSS was an independent risk factor for OS and CSS. However, from the 15th year after surgery, the CSS of the FSS group began to significantly decrease compared to that of the definitive surgery group. Therefore, FSS is considered for patients with reproductive needs, but the recommended follow-up time is not less than 15 years. In addition, for patients with IC stage disease, FSS should be selected more carefully, and long-term close follow-up is required.

The survival analysis results of stage I ovarian SCSTs have been reported (9–11), but the Fine-Gray competitive risk model has not been evaluated. In these studies, patients with competitive events were listed as censored data, so the results were biased to different degrees. Therefore, we adopted the Fine-Gray model to explore the influence of FSS and other factors on patient prognosis.

We found that FSS was more likely to lead to worse CSS than definitive surgery. We also found that, in the first 10 years after surgery, the CSM and CSS in the two groups were similar; however, from the 15th year after surgery, the CSM of the FSS group was higher than that of the control group. At the 20th and 25th years postoperatively, the CSM of patients who underwent was approximately twice that of the control group. This phenomenon may be related to the cohort characteristics in the present study. A retrospective study involving 160 patients with stage I granulosa tumor found that patients with stage IC had a higher recurrence rate (43% vs 24%) and shorter median recurrence time (10.2 vs 16.2 years) than those with stage IA (13). In our study, the proportion of patients with IC in the FSS group was 24.19%, which was higher than that in the control group (17.24%). The long-term CSS in the FSS group was significantly lower than that in the control group, which may be related to the worse prognosis caused by the long-term recurrence of IC stage tumors. Simultaneously, we found that patients in the FSS group were younger than those in the definitive surgery group, and the proportion of patients in the 5–34 years group was higher (58.87% vs. 12.07%), whereas the proportion of patients in the 35–49 years group was significantly lower than that in the control group (41.13% vs 87.93%). The older one gets, the higher the overall risk of disease or death (14). It may be due to the fact that more patients who underwent definitive surgery died of non-ovarian cancer. Studies have found that the size of SCSTs is significantly related to the risk of disease recurrence. For every 1-cm increase in tumor size, the risk of recurrence increased by 20% (HR, 1.20; 95% CI, 1.11–1.07) (15). In the FSS group, there were fewer people with tumors ≤ 100 mm (33.06% vs 43.97%) and more people with tumors between 101 and 200 mm (35.48% vs 25.86%) than in the control group. The number of patients with tumors > 200 mm was the same (11.29% vs 11.21%). It can be seen that patients in FSS group have larger tumors, which may lead to higher recurrence rate and worse long-term CSS.

It is also possible that FSS causes the CSM of patients with stage I SCSTs to gradually increase, leading to a significant decrease in long-term CSS. SCSTs are characterized by late recurrence, and the mortality rate of SCSTs after recurrence is as high as 70% (4, 15). Moreover, the recurrence rate of stage IA ovarian Sertoli-Leydig cell tumors after FSS is 8% and that of definitive surgery is 6% (16). FSS refers to unilateral or bilateral salpingo-oophorectomy (uterus preservation), while definitive surgery includes bilateral salpingo-oophorectomy and hysterectomy. It may be that some patients who underwent FSS retain part of their ovaries and fallopian tubes but omit the small lesions in these tissues. It is also possible that these tissues provide soil for the recurrence of SCSTs and reduce the long-term CSS. However, a detailed medical history of patients who died of SCSTs is unavailable in the SEER database, which is only speculative and cannot confirm the specific reason.

In our study, definitive surgery was found to have no significant advantage in prolonging the OS and CSS within 15 years after surgery. From the 15th year postoperatively, the CSS of the FSS group began to significantly decrease compared with the control group, and the CSS continued to decrease 20 years postoperatively, but tended to be stable at the 25th year. Furthermore, SCSTs may have late recurrence 30 years after the initial diagnosis and treatment (6, 17). This differs from our results. The follow-up period of this study was mainly < 240 months, and the number of patients who had > 240 months accounted for only 11.25% of the total. The average follow-up time was 100.85 ± 6.20 months in the FSS group and 143.09 ± 8.42 months in the definitive surgery group. By screening the patients, we determined that the year of diagnosis was between 1988 and 2017. Therefore, the follow-up data of patients aged ≥ 30 years after surgery are insufficient. This needs to be further studied after prolonging the follow-up time.

The results of the univariate analysis of the Fine-Gray model and Kaplan-Meier analysis showed that there was no significant difference in CSS between the two models, indicating that the prediction results of the two models were stable. According to the Kaplan-Meier analysis, CSS was approximately 92% at 10 years and 75% at 15 years after FSS. According to the Fine-Gray model, CSS was approximately 97% at 10 years and 83% at 15 years after FSS, which was higher than that in the Kaplan-Meier analysis. Since the Kaplan-Meier analysis did not exclude the influence of competitive events and included patients who died of non-ovarian causes, the CSS would be underestimated. Using Fine-Gray model for survival analysis can eliminate the impact of competitive events on the prognosis and is a more effective survival analysis model (18, 19).

In addition, the results of our study suggest that there are significant differences between the Fine-Gray and Cox models in predicting independent risk factors for CSS. The Cox model showed that the histological type was an independent risk factor for CSS, whereas the surgical methods had no effect on CSS. However, the results of the Fine-Gray model showed that FSS could lead to higher CSM and lower CSS, and other variables had no significant correlation with CSS. In the Cox model, the endpoints of survival outcomes were divided into two categories: events of interest and censored events (17). However, in the real world, competitive events may block or reduce the probability of the occurrence of events of interest. If competitive events are ignored, the results of the Cox model will be biased (17, 20, 21). Thus, the results show that the Fine-Gray model is more accurate than the Cox model.

This study has some limitations. First, the SEER database does not provide relevant data about the environment and lifestyle and lacks detailed information on adjuvant treatment and auxiliary examination results. Further studies of these variables may affect the accuracy of the prediction model. The chemotherapy scheme and indications for patients with stage I SCSTs are still controversial, and inconsistent treatments may have a certain impact on the prognosis. Finally, the characteristics of the retrospective study may lead to certain biases, for example, the patients in FSS group are younger than those in definitive surgery group because younger patients deserve the option of FSS compared to the older ones. A large-scale prospective study is needed.

In conclusion, FSS is considered for patients with stage I SCSTs who have fertility requirements; however, the follow-up time is recommended to be not less than 15 years, especially for patients with IC stage. This may be a way to improve the long-term survival rate by undergoing definitive surgery after childbirth.

## Data availability statement

The original contributions presented in the study are included in the article/Supplementary Material. Further inquiries can be directed to the corresponding author.

## Author contributions

JF conceived and designed the study. DS drafted the manuscript, produced the figures and tables. ZZ analyzed the data and formatted the article. All authors contributed to the article and approved the submitted version.

## Funding

This study was supported by the Natural Science Foundation of Guangxi Province (grant no. 2020JJB140152), the Self-funded project of Guangxi Health Commission (grant no. Z20200232), the Key Research and Development program of QingXiu District Science and Technology plan (grant no. 2020052), the Health Appropriate technology Development and Promotion project of GuangXi (grant no. S2019103), and the Guangxi Medical and Health Appropriate Technology Development and Promotion And Application Project (grant no. S2018004).

## Conflict of interest

The authors declare that the research was conducted in the absence of any commercial or financial relationships that could be construed as a potential conflict of interest.

## Publisher’s note

All claims expressed in this article are solely those of the authors and do not necessarily represent those of their affiliated organizations, or those of the publisher, the editors and the reviewers. Any product that may be evaluated in this article, or claim that may be made by its manufacturer, is not guaranteed or endorsed by the publisher.
